# Trajectory Analysis in Single-Particle Tracking: From Mean Squared Displacement to Machine Learning Approaches

**DOI:** 10.3390/ijms25168660

**Published:** 2024-08-08

**Authors:** Chiara Schirripa Spagnolo, Stefano Luin

**Affiliations:** 1 NEST Laboratory, Scuola Normale Superiore, Piazza San Silvestro 12, I-56127 Pisa, Italy; 2 NEST Laboratory, Istituto Nanoscienze-CNR, Piazza San Silvestro 12, I-56127 Pisa, Italy

**Keywords:** particle dynamics, molecular diffusion, molecular trajectory statistics, single-molecule analysis, single molecule tracking, machine learning in biology, quantitative microscopy, quantitative biology, hidden Markov models, moment scaling spectrum

## Abstract

Single-particle tracking is a powerful technique to investigate the motion of molecules or particles. Here, we review the methods for analyzing the reconstructed trajectories, a fundamental step for deciphering the underlying mechanisms driving the motion. First, we review the traditional analysis based on the mean squared displacement (MSD), highlighting the sometimes-neglected factors potentially affecting the accuracy of the results. We then report methods that exploit the distribution of parameters other than displacements, e.g., angles, velocities, and times and probabilities of reaching a target, discussing how they are more sensitive in characterizing heterogeneities and transient behaviors masked in the MSD analysis. Hidden Markov Models are also used for this purpose, and these allow for the identification of different states, their populations and the switching kinetics. Finally, we discuss a rapidly expanding field—trajectory analysis based on machine learning. Various approaches, from random forest to deep learning, are used to classify trajectory motions, which can be identified by motion models or by model-free sets of trajectory features, either previously defined or automatically identified by the algorithms. We also review free software available for some of the analysis methods. We emphasize that approaches based on a combination of the different methods, including classical statistics and machine learning, may be the way to obtain the most informative and accurate results.

## 1. Introduction

Single-particle tracking (SPT) is an established technique for observing the behavior of single entities at high spatial and temporal resolution (nanometers and milliseconds) in various scientific fields such as Biology, Chemistry, and Physics. SPT is based on the reconstruction of the trajectories of single particles visualized in real time in the system of interest. In life science, for example, it has been applied to resolve the working mechanisms of molecules, organelles, and viruses [1,2,3,4].

The technique requires synergistic efforts in several aspects, such as instrumentation, particle labeling, and data analysis [5,6,7,8,9]. Experimental time-lapse images are processed with two main analysis steps—trajectory reconstruction and trajectory analysis [1,10]. The latter allows for the extraction of various parameters characterizing the behavior of the tracked particles, such as the type of motion, diffusion coefficient, velocity, and/or interaction events thus finding the crucial links between the observed motions and the processes driving them. Over the years, different types of possible trajectory analyses have been developed, with the aim of extracting more and more detailed information about the mechanisms underlying the observed trajectories. With the proliferation of analysis methods, it is useful to have some overview of the different possibilities to identify approaches that may be useful for the application of interest. The available reviews on SPT typically aim to cover all the aspects of the technique, often including its applications [1,9]. Here, we present a comprehensive review focusing on the last step of the technique, i.e., track analysis.

First, we briefly review the most common and traditional methods of track analysis, which are based on the mean squared displacement (MSD) function. In particular, we highlight some factors that are not always considered but can affect the precision and accuracy of the results, especially the choice of the number of points to consider in the MSD curve for extracting parameters like the diffusion coefficient and the effects of localization uncertainty, as well as of some experimental parameters such as temporal resolution. MSD analysis is challenged by measurement uncertainties and by the presence of too-short trajectories and of heterogeneities, particularly in the case of anomalous motion [11,12]. It remains a valid tool, but various studies have indicated that alternative and sometimes complementary approaches might allow for the obtaining of more informative results. Distributions of parameters other than displacements have been considered, e.g., the angular distribution within tracks, which has proved to be more sensitive for quantifying caging and distinguishing different and even rare transport mechanisms [13].

Due to environmental heterogeneities, the presence of interactions or other processes, and changes in motion type and parameters can also occur within a single trajectory [14]. This demands methods to partition the trajectory into multiple segments and to characterize the motion in each state and the switching behavior between them. In some cases, the presence of multiple states within single trajectories was undetectable in an MSD analysis; it was instead revealed by more advanced approaches, which allowed for the characterization of multiple states with their switching kinetics, and the consequent construction of models of the underlying mechanisms [15,16]. Hidden Markov model approaches have been applied in some cases to classify states characterized by different diffusivities or types of motion and to extract populations and switching probabilities. We discuss the limitations and challenges of this kind of analysis as well; these are related to the definition of the states in the case of non-pure Brownian motion and to the choice of the number of states to be considered.

Nowadays, classification is one of the main tasks solved by machine learning, which has therefore also been applied to trajectory analysis. In the last part of the review, we focus on these kinds of methods, which have greatly expanded in recent years. We describe approaches based on several machine learning methods, from random forest to deep neural networks. Machine learning methods are demonstrating good accuracy and sensitivity and are proving to be very advantageous for detecting hidden phenomena and extracting valuable results even from short and noisy trajectories. However, also in this case, several challenges have arisen, such as transferring learning to experimental data after training on simulations, time, and computational requirements, and interpretability and explainability. We focus on some examples that show how the integration of classical statistics can be very helpful in assessing the validity of the results and in defining the best set of features used by the machine learning algorithm to classify trajectories.

## 2. Mean Squared Displacement (MSD) Analysis

**Figure 1:**
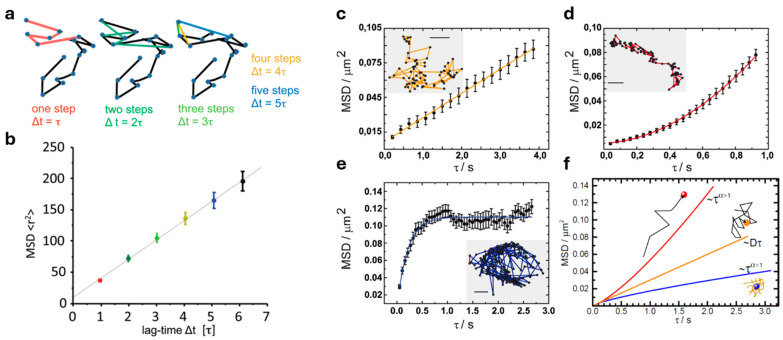
Mean Squared Displacement (MSD) analysis. (**a**) Different numbers of steps (different time lags) are considered for the MSD calculation. (**b**) The average of all displacements with the same time lag gives a point in the MSD curve. (**c**–**e**) The plot of MSD versus the time lag (τ) allows the classification of the type of motion for the trajectories (in the insets) showing Brownian (**c**), drifted (**d**), and confined (**e**) motion. (**f**) Examples of MSD behavior for superdiffusive (red line) and subdiffusive (blue line) “anomalous” motion, compared with the one for a Brownian trajectory (orange line). Bar in (**c**–**e**) insets: 0.16 μm. Panels (**a**,**b**) are adapted with permission from [3] © 2011 The American Society of Gene and Cell Therapy, published by Elsevier Inc. Panels (**c**–**e**) are adapted with permission from [18] © 2011 Elsevier B.V.

Mean Squared Displacement (MSD) is the most common analysis in an SPT study. It is the second moment of the displacements distribution as a function of time lag, i.e., it quantifies the average squared distance traveled by a particle in a certain time (Figure 1a,b). It can be calculated as follows:

$$MSD\;\left(\tau =n\Delta t\right)\equiv \frac{1}{N-n}\sum_{j=1}^{N-n}{|X\left(j\Delta t+\tau \right)-X\left(j\Delta t\right)|\;}^{2},$$

where N is the number of points in the trajectory $X\left(\tau \right)$, the latter being sampled at times Δt, 2Δt,… NΔt and $\left|\ldots \right|$ represents the Euclidean distance. To be more specific, the reported calculation is used to estimate the time-averaged MSD (TAMSD) for each single-particle trajectory; this is the most used formulation. Another possible calculation is the so-called ensemble-averaged MSD, in which the displacements are averaged over multiple particles at time t. The first one is preferred because of possible heterogeneities in a population of different particles, but tracks of sufficient length are not always available. The two averages can also be combined into the time- and ensemble-averaged mean-squared displacement (TEAMSD) [17].

The MSD function can reflect the kind of particle motion. It has been used to distinguish between immobile, pure Brownian, confined and directed motions (with the latter usually having a component of Brownian diffusion and a component of active transport with a constant drift velocity), because these show different trends in their associated MSD [18,19,20] (Figure 1c–e). For Brownian motion, the MSD increases linearly with time, whereas linearity is lost for the other types of motion. The expressions used to fit the MSD function for the different motions are well established and can be found elsewhere [21].

For each kind of motion, fitting the MSD function leads to the extraction of parameters describing the specific situation, e.g., the diffusion coefficient D for the Brownian motion, velocity and diffusion coefficient for the directed motion, confinement radius, and characteristic time for the confined motion [9].

In some cases, such as molecular labeling with organic dyes subject to photobleaching, the trajectories are relatively short and only the initial part of the MSD curve can be reconstructed, making it difficult to capture the nature of the movement from the MSD fit [14]. In these situations, and when one wishes to measure a diffusivity regardless of the kind of motion, the slope of a straight line passing through the first MSD points is used to calculate a short-term diffusion coefficient [2,18,20,21,22,23].

The MSD function of a trajectory in ν dimensions can also be fitted with a general law as follows [24,25]:

$$MSD\left(\tau \right)=2\nu D_{\alpha }\tau^{\alpha }$$

where $D_{\alpha }$ is the generalized diffusion coefficient (or anomalous diffusion constant) and α is the anomalous exponent (Figure 1f); α close to 1 characterizes Brownian motions, α < 1 corresponds to subdiffusion, α > 1 corresponds to superdiffusion. A log–log plot of MSD versus time is commonly used, where α is the slope of the curve in such plot [26].

A precise determination of the scaling exponent when the type of motion does not change in the single trajectory would require at least two order of magnitude for the time lags at which the MSD is calculated; however, some insights on the type of motion can be inferred also from estimates calculated by considering a lower number of initial points, especially in the case of non-homogeneous motion, e.g., for demonstrating that a trajectory can have several drifted subtrajectories, even if it seems Brownian or even confined at longer lag times [27].

Estimates of the standard diffusivity D (from a linear fit of MSD versus lag time) and α are often used together to assign the type of motion. For example, Jobin et al. performed an SPT study on GABA_B_ (γ-aminobutyric acid type B) receptors and classified the trajectories with D < 0.01 μm^2^ s^−1^ as immobile; the trajectories with D ≥ 0.01 μm^2^ s^−1^ and 0.75 ≤ α ≤ 1.25 as Brownian diffusive; the trajectories with D ≥ 0.01 μm^2^ s^−1^ and α < 0.75 as sub-diffusive, the trajectories with D ≥ 0.01 μm^2^ s^−1^ and α > 1.25 as super-diffusive [28].

Anomalous diffusion is a phenomenon often found in SPT performed in different contexts in live cells, from membranes to the cytoplasm and nucleus; this kind of motion has multiple origins, such as crowded environments, presence of interactions, different transport mechanisms; deciphering its nature and causes is very helpful in understanding fundamental cellular processes [9,26,29,30,31,32]. For the descriptions of anomalous motion models, we refer to [9,33,34,35].

One area where the MSD analysis of single-molecule trajectories has been widely applied concerns the study of membrane receptors in living cells. From the work of Kusumi et al. [19], this approach has been used to characterize the kind of motion of different receptor families, e.g., epidermal growth factor receptors [19,36], transferrin receptors [19], neurotrophin receptors [18], and G protein-coupled receptors [37,38,39]. Typically, four types of motion (stationary, Brownian, directed, confined) have been identified thanks to MSD analysis, and the changes in their population under different conditions have provided insight into the correlation between the type of motion and the function performed by the molecule [18,37,38,39]. The presence of different types of motion has played a fundamental role in defining the models of the plasma membranes, e.g., this approach helped to demonstrate the existence of membrane compartments associated with the cytoskeleton network [19].

However, caution must be taken in the MSD analysis as some papers have pointed out the influence of experimental conditions (especially those related to uncertainties) on the accuracy and reliability of this analysis and of the extracted motion parameters, as we will discuss below.

It has been reported that, in the case of Brownian motion, the localization uncertainty (the so-called static error) produces a positive offset in the MSD curve, and the finite camera time exposure (the so-called dynamic error) produces a negative offset; considering both contributions, the MSD function for a ν–D trajectory can be written as follows [40,41,42]:

$$MSD(\tau )=2\nu D\tau +2\nu \sigma^{2}-4\nu DR\Delta t$$

where D is the diffusion coefficient, $\sigma^{2}$ is the variance of the position coordinates for an immobile particle, R is the motion blur coefficient (which depends on the temporal illumination profile during the integration time, e.g., it is equal to 1/6 in the case of continuous illumination), and the other quantities are defined as above [40,41,42].

Static and dynamic errors must also be considered for other kinds of motion, but the formalization of their contributions is more controversial in cases other than the Brownian one [43]. Backlund et al. showed that both static and dynamic errors must be taken into account in the case of anomalous diffusion and derived an analytical expression for the MSD that includes both these errors in the case of fractional Brownian motion (FBM) [43]; FBM is a generalization of the Brownian motion such that the distribution ${\tilde{B}}_{H}\left(t\right)$ of the displacement at time t is Gaussian with variance proportional to $t^{2H}$, it is self-similar (i.e., ${\tilde{B}}_{H}\left(\alpha t\right)={\alpha^{H}\tilde{B}}_{H}\left(t\right)$) and the increments are stationary (i.e., they depend only on the lag time and not on a possibly different starting time) [43]. Destainville et al. calculated the effects of time averaging in the case of confined diffusion and provided a corrected expression for the MSD; however, they showed that when the parameter L^2^/12D (equilibration time, where L is the apparent confinement domain size) is large compared to the exposure time, the estimates of the confinement size and the diffusion coefficient are not affected by the corrections [44].

One question that may arise concerns the choice of the points in the MSD function, and therefore their optimal number, which should be used to extract the estimates of the motion parameters. Indeed, at large time lags, the MSD points are less averaged and thus subject to greater statistical fluctuations, and their cross-correlations are higher than for short time lags (even if these are never zero), therefore they cannot be approximately considered independent measurements; however, at shorter time lags, the MSD points are more affected by the contribution of the localization error [45,46]. The number of points used to estimate the short-term diffusion coefficient was different in different studies, depending on the specific conditions [47,48]. Two points (D_12_, using time lags 1 and 2) were typically used by some groups [2,20,49,50], three points (D_2–4_, time lags 2, 3, and 4) were used by other ones [51,52]. The optimal number of points to use was associated to the so-called reduced localization error $r=\frac{\sigma^{2}}{D∆t}$. Michalet found, through theory and simulations, that when r≪1 (uncertainty dominated by diffusion), the best estimate of D is obtained by considering the first and second points of the calculated MSD (without considering a (0,0) point); when r≫1 (uncertainty dominated by localization uncertainty), a larger number of points are needed to reduce the impact of localization uncertainty, and their optimal number depends on r and on the number of time steps in the trajectory [46]. Ernst et al. analyzed this issue starting from a very long 3D experimental trajectory of a fluorescent bead acquired using orbital tracking and analyzing subtrajectories with fixed numbers of time points extracted from it. They found out that they needed to exclude the MSD point at the shortest time lag because of the impact of residual oscillations of the piezo used in their orbital tracking setup [45]. Considering this experimental quirk, they demonstrated that using four points, i.e., from the second to the fifth point of the MSD, minimized the ratio of the standard deviation over the estimated value of the diffusion coefficient, for a value of r≈0.8, in good agreement with the work of Michalet cited earlier in this paragraph [45]; however, they found that, in their case, the optimal number of MSD points did not seem to depend on the length of the tracks [45].

Kepten et al. published a guideline for MSD analysis in the case of anomalous diffusion [12]. They analyzed the precision and accuracy of the fitted MSD by simulating different conditions in terms of trajectory length, measurement error, and anomalous exponent. In each case, they identified a maximum lag time to be used in the MSD fit that depends on the three parameters. Thus, they concluded that estimating the measurement uncertainty and the range of the anomalous exponent beforehand can significantly help to improve the accuracy of the results, by selecting the optimal number of points for MSD analysis and fitting. Under conditions like short trajectories and no estimation of the measurement uncertainty, or in the case of relatively large measurement uncertainty, the MSD results were highly inaccurate.

**Figure 2:**
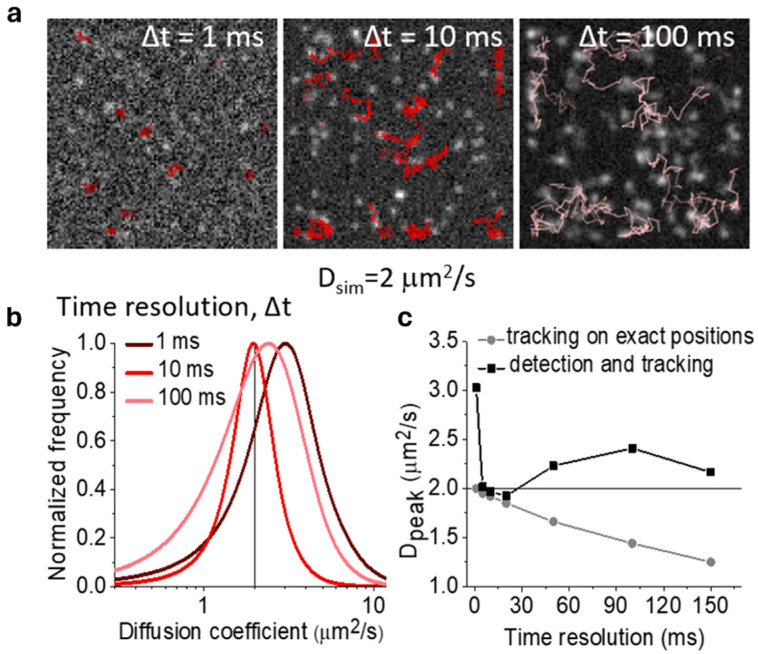
Effects of time resolution on estimates of diffusion coefficient by MSD analysis. (**a**) Movies of single molecules diffusing with Brownian motion are simulated with a diffusion coefficient of 2 μm^2^/s at different time resolutions Δt; we show the first frame of each movie and some of the reconstructed trajectories. (**b**) The histogram shows the distributions (normalized to 1 at the peak) of the short-term diffusion coefficients estimated from the first two points of the MSD function using three different time resolutions. Data were obtained by detection and tracking on simulated movies. (**c**) The peak of the estimated distribution of the short-term diffusion coefficient (D_peak_) is shown at different time resolutions. Data were obtained by detection and tracking on simulated movies (black, including static and dynamic localization errors and tracking errors) and by tracking on exact simulated positions (grey, including tracking errors only). See [53] for more details and examples.

We have also previously investigated the effects of different uncertainties and errors on MSD-based estimates [53]. In particular, we considered the effects of different temporal resolutions on the distribution of the short-term diffusion coefficient in the case of pure Brownian motion (Figure 2). We observed that variations in temporal resolution can cause shifts and broadening in such distribution. This type of effect is related to both localization uncertainty (static and dynamic, the latter producing motion blur effects) and tracking errors, which have different impacts on the diffusivity estimates depending on the interplay of temporal sampling with conditions such as diffusivity, density, signal-to-noise ratio. We showed that the observed effects cannot be fully explained by the available theory, e.g., the additive offset introduced in the MSD function to include static and dynamic errors [40,41,42] discussed above is not enough to explain the effects we observed. Also, in this case, preliminary evaluations of the impact of measurement and analysis uncertainties are helpful in choosing the proper experimental conditions, such as the temporal resolution, and to improve the accuracy and precision of the results.

In addition to the caveats and necessary precautions discussed above, it is also important to note that a simple MSD analysis is not sufficient in many cases; it cannot be used when multiple modes of motion compose a single trajectory, and it also cannot discern amongst different mechanisms producing the same MSD behavior, especially in the case of anomalous diffusion. We will describe these cases in the following sections, and discuss some alternative methods for analyzing the trajectories derived from SPT.

## 3. Alternatives to MSD Based on Classical Statistics

An often-used alternative to the MSD analysis is the Moment Scaling Spectrum (MSS); while MSD focuses only on the second moment of the particle position distribution, the MSS considers more moments of order ν for different time lags τ as follows [54]:

$$\mu_{\nu }\left(\tau \right)=\frac{1}{N-n}\sum_{j=1}^{N-n}{\left|X\left(j∆t+\tau \right)-X(j∆t)\right|}^{\nu }.$$

**Figure 3:**
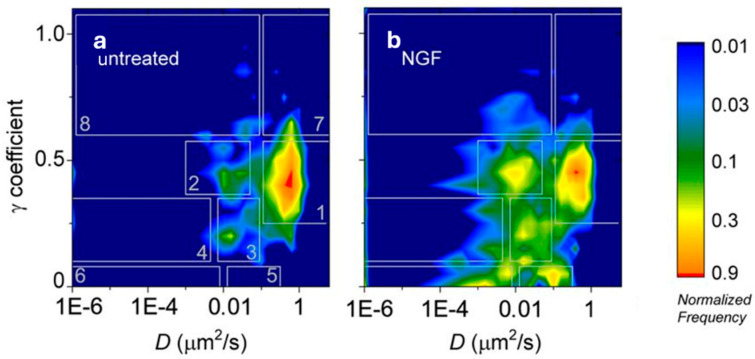
Bidimensional histograms of the γ coefficient (from the MSS analysis) versus the short-term diffusion coefficient D (from the MSD analysis) measured by SPT for the TrkA receptor (unstimulated in (**a**), stimulated by the nerve growth factor, NGF, in (**b**)). White rectangles numbered from 1 to 8 correspond to the different identified dynamic regions [23]. On the right the color bar shows the frequency of the total D-γ distributions in logarithmic scale and normalized to 1 at the peak. Reproduced with permission from [23] (© 2013 The Company of Biologists Ltd.), permission conveyed through Copyright Clearance Center, Inc.

The case ν=2 is the MSD, and usually ν from 1 to 6 are considered [55,56]. Each moment is assumed to depend on the time lag in the form $\mu_{\nu }(\tau )\sim \tau^{\gamma_{\nu }}$ eventually, with an addictive offset to include the effects of uncertainties explained above. The plot of $\gamma_{\nu }$ versus ν is called the moment scaling spectrum (MSS). By analyzing the MSS, one can understand if there could be distinct modes of motion in a single trajectory. Indeed, for a self-similar trajectory, the MSS can be fitted by a straight line passing through the origin and its slope γ reflects the type of motion as follows: a slope close to zero characterizes stationary particles; a slope between 0 and 0.5 indicates a sub-diffusive/confinement regime; a slope close to 0.5 indicates pure diffusion; a slope higher than 0.5 characterizes the super-diffusive regime (γ gives the same classification as half the anomalous exponent α defined above, and the value should also be almost the same for self-similar trajectories) [55,56,57]. In this case, since the MSS is based on more moments than the MSD, it can provide a more robust characterization of the motion. If, instead, the $\gamma_{\nu }$ is not directly proportional to ν, one can conclude that the trajectory is not self-similar and suspect of inhomogeneities in the particle motion; in this sense, MSS is more useful in analyzing complex and dynamic motion patterns [57]. When inhomogeneous trajectories are suspected, they are then often subjected to a segmentation in subtrajectories, e.g., detecting transient arrests of diffusion (TADs, a method based on transient confinement analysis as described in the following and in [58]). This kind of analysis has been used for many SPT analyses [23,54,56,57,59,60]. Marchetti et al. used the MSS analysis to distinguish between self-similar and multimodal trajectories in an SPT study on the neurotrophic receptor TrkA [23,60]. By combining MSS, MSD, and TAD [58] analyses, they obtained a classification of trajectories and subtrajectories into immobile, slow, fast, drifted, and TAD events. Moreover, they used bidimensional histograms of γ versus D_12_ (the short-term diffusion coefficient obtained by MSD, Figure 3) measured for the receptor under different conditions (stimulation by different ligands or administration of different drugs to the cells), identifying different dynamic regions in the plot. An analysis of the changes in populations in these regions in the different conditions allowed for the association of each region with a behavior of the receptor (such as free-diffusion, formation of signaling platform, assembly of signaling endosomes precursors). The authors concluded that the receptor dynamics on the plasma membrane is a specific signature of its activation state and is ligand-dependent (different ligands produce different dynamics patterns called the “ligand fingerprinting” effect); therefore, the receptor dynamics induced by ligand binding has a strict correlation with the induced biological response.

An important type of motion for biological SPT applications is the one caused by confinement; considering membrane proteins for example, this can be due to clustering, to cytoskeleton-mediated compartmentalization, or to the presence of lipid domains, and is often associated with the activation of receptors or the regulation of their density in specific areas [23,61,62,63]. Indeed, one of the first methods to detect changes in motion within trajectories focused on the detection of transient confinement. Such periods of motion were identified by calculating the probability of remaining in a region for a significantly longer period of time compared to the case of a pure Brownian motion with the average D of the trajectory. The analysis was performed by testing the average square displacement per frame of a particle in a moving window and allowed for the separation of a track into different segments [58]. This method has been widely applied and continues to be so [23,64,65,66]. There are also other more general approaches based on the segmentation and classification applied on different rolling windows within tracks, for example, using MSD analysis in each window [67,68] or considering velocity statistics (e.g., average, median, and/or maximum, considering its direction as well) in each window [27,69,70,71]. One challenge with this type of strategy is the choice of the window length; indeed, smaller windows are required to increase the sensitivity for the detection of motion switches, but larger windows are required to increase the accuracy of motion classification [67,72]. Thus, in some cases, this type of approach fails to detect short-lived motion types or gives variable results depending on the window size [23,73].

Vega et al. developed a method called the “divide-and-conquer moment scaling spectrum” (DC-MSS), which uncouples the detection of motion switches from motion classification, overcoming the main limitation of traditional rolling-window-based approaches [72]. After a first segmentation based on a local movement descriptor (the maximum pairwise distance between particle positions within an 11-frame moving window), the motion classification is performed via the MSS analysis, which is also exploited to refine the initial track segmentation. One reported limitation was the requirement of relatively long periods (at least 20 frames) characterized by the same type of motion for correct identification. However, the method was tested on the cell membrane protein CD44 tracked in macrophages and was able to distinguish changes in mobility due to interactions with the actin cortex [72].

Another possible analysis approach is based on the van Hove correlation (vHc) function, which is the displacement distribution at the lag time of interest [13,74]. For pure or fractional Brownian motion, the van Hove correlation function is a Gaussian distribution. The Gaussian form of the ensemble-averaged vHc function indicates a homogeneous system of particles moving with a Brownian motion and the same diffusion coefficient. Deviations from Gaussian behavior indicate heterogeneous systems (in the case of an ensemble-averaged distribution) or non-Brownian motion in the case of a single-particle displacement distribution. In a study of transcriptional regulators, Wagh, Stavreva et al. approximated the vHc as a superposition of Gaussian functions and, using an iterative inversion procedure, calculated the distribution of MSDs, finding two different mobility states in single nucleosome tracks [75].

Spot On is a free tool for SPT analysis based on the fitting of an analytical model to the empirical displacement distribution at various time lags (equivalent to the vHc) using non-linear least squares fitting [76] (Table 1). The model is constructed by calculating the probability of a particle having a given displacement in a given time delay; it accounts for localization errors (which can be user-defined or inferred from the data) and corrects for the defocalization bias (out-of-focus motion) and the undercount of the fast diffusing population (caused by the fact that such molecules spend less time in focus and are more subject to motion blurring). The model considers only pure Brownian motions and two or three possible populations. Spot On extracts diffusion coefficients and subpopulations of trajectories. It has been tested and validated on simulated and experimental SPT data; the latter obtained by tracking different types of nuclear protein dynamics in live cells. The Spot On web interface is freely available at [77]; the code is available on GitLab [78]; the Matlab command line version is available at [79]; and the Python command line version is available at [80]. A TrackMate plugin is also available which allows the trajectory obtained by TrackMate to be uploaded directly to Spot On for analysis [81].

Heckert et al. developed a method based on Bayesian analysis that they refer to as state array SPT (SaSPT, Table 1) [82]. It approximates continuous diffusion coefficient distributions with a grid of discrete states to calculate the posterior occupancy at each point in the grid. The method can estimate an unknown number of diffusion states, even in the case of non-discrete diffusion coefficient distributions, incorporates localization errors, and corrects for the overestimation of the population of slow states due to the defocalization bias. However, it does not deal with transitions between states. SA can be generalized to any motion model parameterized by a likelihood function. The method has been implemented in a Python module available on GitHub [83], with available documentation [84].

**Figure 4:**
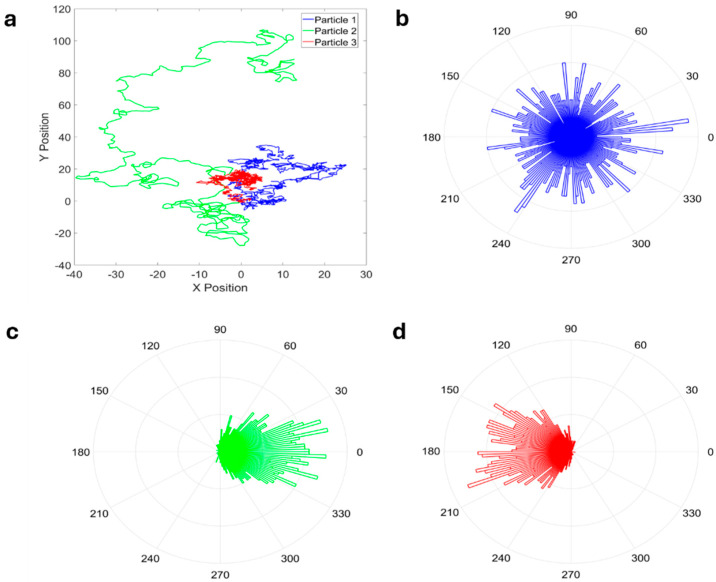
(**a**) The trajectories of three particles are shown as examples in blue, green, and red. Panels (**b**–**d**) show the radial histograms obtained by calculating the angular displacement between each successive time step: the angle is reported along the azimuthal axis, the frequency for that angle is reported along the radial direction. In (**b**), the particle has no angular preferences, typical of Brownian motion; in (**c**), the particle tends not to change direction (angular distribution center close to 0°); in (**d**), the particle tends to move in the opposite direction for each time point (angular distribution center close to 180°). Reproduced with permission from [14] (© 2019 John Wiley and Sons, Inc.), permission conveyed through Copyright Clearance Center, Inc.

Several other approaches, typically based on the distributions of the parameters other than displacements, have been proposed to increase the amount of information extracted from trajectories, particularly to gain more insight into the underlying mechanisms governing the observed system [13]. A relevant example is the distribution of directional changes within trajectories [13,94,95]. Burov et al. considered relative angles at different time scales, where the trajectories were sampled with a certain time resolution, the resulting points were connected by vectors, and the angles between successive vectors were measured. Relative angles have been shown to have different distributions for different types of motions, and, by using variable time intervals, it is possible to capture the time scale over which the kind of dynamic is relevant [94]. Distribution of angular displacements can be plotted using radial histograms (Figure 4). As observed by Harrison et al., the angular distribution and the MSD analysis can be used as complementary approaches; in this way, the authors analyzed the motion of lipid droplets in living cells and were able to identify different motion regimes (characterized by different angular persistence and different features of sub/super-diffusivity) at different time scales [95], with transition points between the regimes that coincide in the two types of analysis [95]. The determination of directional persistence and associated time-dependent directional changes have been performed by analyzing the distribution of a turning angle between the segments of the track as a function of the lag time [13,14]. Directional persistence may be indicative of an active transport mechanism driven, for example, by molecular motors [13,14].

Another quantity used in the analysis of transport mechanisms is velocity. The distribution of velocity within individual particle trajectories has been used, for example, to characterize the motion of the motor protein Myosin V in the cytoplasm of live cells [96], the axonal transport of vesicles carrying neurotrophins in neurons [69,70,71] (with the used MATALB scripts reported in [71]) or the endocytic pathway of the influenza virus [97]. Tejedor et al. proposed a method based on a mean maximal excursion statistic (MMEs) for the analysis of different kinds of anomalous subdiffusion motions [98]. The maximal excursion is the largest distance covered by a particle in a time interval; an average is performed over all the trajectories. They showed that MSD analysis is not able to discriminate between two models used to simulate different kinds of anomalous diffusion that give rise to the same anomalous exponent; instead, the additional observables they introduced, based on the moments of the MME, allowed them to distinguish between different subdiffusion mechanisms as well as to obtain more precise estimates of the anomalous exponent [98,99].

Also, Condamin et al. [100] observed that subdiffusion can arise from different mechanisms leading to similar scaling laws, which can therefore not be distinguished by MSD analysis alone. The authors considered the following two different microscopic models of subdiffusion: (i) continuous time random walks (CTRWs) with a heavy-tailed distribution of waiting time, where the high waiting times could be caused by a crowded environment or to metastable chemical binding (in “traps”); and (ii) motion with fixed fractal obstacles. These models are considered relevant for anomalous subdiffusive motion in living cells, where crowding can be due to the high density of molecules, aggregates, or “traps”, e.g., in the plasma membrane or in the cytoplasm, and fixed obstacles can be created by membranes in the cytoplasm or cytoskeletal elements in both the plasma membrane and cytoplasm. They based the analysis on three “first-passage observables”: (1) the time it takes a particle from a given site to reach a target for the first time (first-passage time); (2) the probability of reaching a given target before reaching another target (first-passage splitting probability); and (3) the time a particle spends at a given site (occupation time). The distribution of these variables is different in the two subdiffusive mechanisms, providing information on the underlying nature of the processes, and, e.g., allowing for an explanation of the kinetics of reactions especially when they are transport-limited (i.e., in conditions of low concentrations of reactants).

Izeddin et al. studied the target-search strategies of two different transcription factors in the nucleus using SPT [30]. The two molecules were the proto-oncogene, c-Myc, and the positive transcription elongation factor, P-TEFb. The authors showed that these two transcription factors adopted the following different strategies to explore the nuclear space and search for their targets: c-Myc showed free diffusion, more specifically, c-Myc used a “non-compact” strategy, moving everywhere in the nucleus with an equal chance of reaching any target regardless of its position; P-TEFb showed a motion confined by fractal structures, i.e., P-TEFb followed a “compact” behavior in which a specific path guides it to its potential targets. The authors used analysis based on MSD, angular distributions and first-passage observables to support their conclusions, providing an interesting example of how different methods of analysis can be exploited together and complemented to gain more insight into the observed trajectories and to construct more informative descriptions of the processes under investigation. Their results showed that the nuclear architecture is protein-specific, and that the geometry of the exploration paths is important for regulating transcription factor function and controlling gene expression [30].

Other approaches introduced to investigate anomalous motions, e.g., to distinguish between different types of subdiffusion, are based [99,101,102] on the velocity autocorrelation function [33,103,104], on the displacement correlation function [105,106], on the power spectral density of the displacement [107,108,109], on the amplitude scatter distribution (which describes the distribution of the TAMSD around the TEAMSD when considering multiple trajectories/particles) [110,111,112], and on p-variations [101,102]. p-variation for a trajectory $X\left(\tau \right)$ of total duration T is defined as starting from the partial sum of increments as follows:

$$V_{N}^{\left(p\right)}\left(t\right)=\sum_{j=0}^{2^{N}-1}{\left|X\left(\min\left\{\left(j+1\right)\frac{T}{2^{N}},t\right\}\right)-X\left(\min\left\{j\frac{T}{2^{N}},t\right\}\right)\right|}^{p},$$

where t<T and $\left|\ldots \right|$ indicates the Euclidean distance; the p-variation is formally the limit for N→∞ of $V_{N}^{\left(p\right)}\left(t\right)$, but some insight on the process behind a motion type can be inferred by studying how estimates of $V_{N}^{\left(p\right)}\left(t\right)$ statistical changes with the exponent p and with time, especially for subdiffusive motion; in particular, the limit exists, is non-zero and finite, and, on average, tends to the p^th^ momentum of displacement at time t, only for p=2 in the case of Brownian motion and of CTRW, and for p=2/α for a FBM or a random walk on a fractal structure (RWF) with an anomalous exponent α [99,101,102].

Weron et al. recently published a study comparing different analysis approaches on the SPT data obtained on G proteins and G-coupled receptors with the aim of classifying their dynamics [113]. First, they used a standard MSD approach based on the use of thresholds for the anomalous coefficient to define the regions of sub-, normal and super-diffusion. Due to the strong influence of the cutoff values on the results, they turned to statistical hypothesis testing. They used the anomalous exponent as the test statistic in a two-sided approach, testing the null hypothesis of free diffusion against the two alternatives and calculating the critical regions after choosing the significance level. They also applied the statistical testing approach using statistics derived from the maximum excursion (similar to the MMEs) and from p-variations. The classification of trajectories led to different results depending on the used method. The standard MSD approach underclassified free diffusion and overclassified superdiffusion; p-variations is the method that gave the highest percentage of subdiffusion. With the help of simulations, they showed that the results depended indeed on the type of process. The statistical approach was more robust than the standard MSD based on the cut-offs; but none of the tested statistics performed at best in all the cases and each one gave the best result in specific situations (i.e., depending on the kind of motion and length of tracks). They suggested combining the approaches by trying to apply all the methods. If the classification results were compatible, any of the methods could be used; if not, they proposed either to calculate an average of the results, or to use other methods to identify the process driving the movement and applying the most accurate test in such a case.

## 4. Markov Modelling

The task of trajectory segmentation and classification in SPT can be approached by methods based on Markov Modelling, which is used to describe systems that can exist in different states with certain transition probabilities between the different states, where these probabilities depend only on the current state and not on previous ones. In SPT, hidden Markov models (HMMs) are typically used, in which the Markov process is assumed to have unobserved (hidden) states, and the observations made provide indirect information about the state. In the end, HMMs allow for the characterization of different diffusive states and estimating the probabilities of transition between them.

Cairo et al. studied, with this method, the mobility of CD45 in T lymphocytes investigated by SPT [16]. They applied both MSD analysis and HMM analysis. Only HMM could detect heterogeneities and motion transitions within a single trajectory (masked in MSD analysis), extracting the switching kinetics between the two identified states. HMM allowed the authors to obtain the association and dissociation rates for the interaction between CD45 and the cytoskeleton, and then design a model for these interactions.

In several cases, the HMM analysis is applied considering a random switching between (typically two) purely diffusive states distinguished by different diffusion coefficients [114,115,116]. However, some studies have shown that, in certain cases, when there are also non-pure Brownian motion types, such a description of states cannot detect changes in the kind of motion. Monnier et al. observed this problem when considering trajectories that included switching between directed and random motion. To detect direct transport, they had to develop an HMM analysis that explicitly included models of both diffusive and directed motions, with Bayesian selection inferring the one consistent with the displacement along the tracks. With this approach, they were able to detect the transient transport states from the tracks of mRNA–protein complexes in living cells [73].

Slator et al. also found that an HMM approach detecting transitions only as changes in diffusivities is not suitable for segmenting the trajectories of the ganglioside GM1 in model membranes. A specific modeling of confinement was necessary and was incorporated into an HMM analysis based on the switching between free diffusion and confinement in a harmonic potential well, potentially slowly diffusing. The developed model allowed for the detection of the two states, their switching times and their parameters, i.e., the free diffusion coefficient, the strength of the potential well, and the position of the potential well center and its diffusivity [117].

The HMM was also developed for the identification of states with two different directional persistence inside single-particle trajectories, in order to study different intracellular transport mechanisms; the directional persistence on a point was inferred by considering the turning angle around that point at different multiples of the frame time (from one to five, in the analyzed examples) [85]. The authors achieved a good separation into “active” and “inactive” states. However, they needed to classify the turning angles with only two states (forward and backward turns) to keep the number of model parameters low, and they recognized that this modeling was a crude simplification [85].

**Figure 5:**
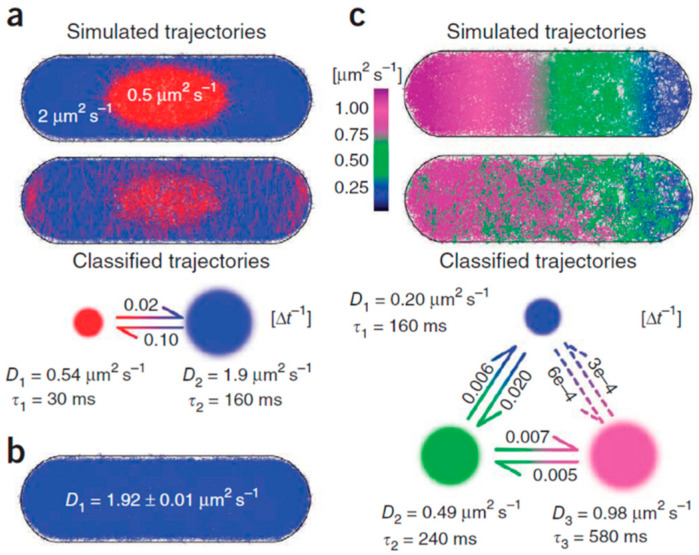
Variational Bayes SPT (vbSPT) software applied to simulated data. (**a**) Top: simulation of a smaller region with lower diffusion coefficient within a larger area with higher diffusion coefficient. Bottom: results of the vbSPT analysis that identified two states described by the indicated parameters of diffusion coefficient (D), mean lifetime (τ) and transition probabilities during one time step (0.02, 0.10). (**b**) A single state is correctly identified by vbSPT for a homogeneous region with a single diffusion coefficient. (**c**) (**Top**): simulation of a region with a continuously increasing diffusion coefficient along the axis. (**Bottom**): results of the vbSPT analysis that identify three states with the indicated parameters. Reproduced with permission from [118] (©2013 Nature America, Inc.), permission conveyed through Copyright Clearance Center, Inc.

One challenge with HMM models is finding an objective way to determine the number of states. This challenge can be tackled using machine learning approaches, as discussed in the next section. Here, we discuss some approaches based on classical statistics. Most of the traditional approaches used a fixed number of states; instead, Persson et al. developed a method (called variational Bayes SPT, vbSPT, Figure 5, Table 1) for HMM-based motion analysis, which inferred the model parameters, including the number of diffusive states, from the data [118]. Each state was described by a diffusion constant and a state lifetime. Identifying the correct number of states required a compromise between the goodness of fit and the complexity of the model. The approach has been applied to the intracellular diffusion of the RNA-binding protein, Hfq, and the different identified diffusive states have been mapped into the corresponding binding states. vbSPT is freely available as a software package [86]. This software has also been used to identify four different states in the mobility of G proteins and their receptors, observed by SPT on the cell membrane [119]. For each state, the authors extracted the diffusion coefficient, occupancy fraction, dwell time, and transition probability from the HMM analysis.

A combined MSS and HMM analysis was performed by Gormal et al. in an SPT study on β2-adrenergic receptors [120]. The MSS revealed a classification of the trajectories into three motion modes—immobile, confined, and free. The HMM confirmed a three-state model (five states allowed; best fit using vbSPT software resulted in three states), which, in this case, was characterized only by different diffusion coefficients (one so low that the state was called “immobile”). This investigation allowed the authors to identify the population of the different states and to study the activation mechanism of the receptor.

Falcao and Coombs also addressed the issue concerning a fixed number of states [92]. They considered a method based on a so-called infinite HMM (iHMM, an extension of HMM to an infinite number of states) which exploits a non-parametric Bayesian approach. It infers the number of states, the transition rates, and the diffusion coefficient defining each state from the data. The software is freely available [93]. As observed for other HMM models cited above, only a pure diffusive motion with different diffusion coefficients was considered; further improvements could include other motion kinds such as confinement or directed motion.

One limitation of HMMs arises from their main characteristic of being memoryless, and this approximation could be too crude in some SPT applications, e.g., for anomalous motion in viscoelastic media, or diffusion in crowded and/or heterogeneous environments [121,122,123]. Indeed, there have been some theoretical or computational works about including memory effects of the (generalized) Langevin equation to study particle motion in complex environments [121,124,125,126]; also, some expected statistics have been calculated in these cases (e.g., MSD and first passage times [122,123]). However, we did not find applications of these methods to the analysis of experimental or simulated single-particle tracks. Although some memory effects in the motion are included in some models of anomalous diffusion already discussed above, we hope that these findings will soon be considered in some automated algorithm for single-particle tracking analysis.

## 5. Machine Learning Analysis

**Figure 6:**
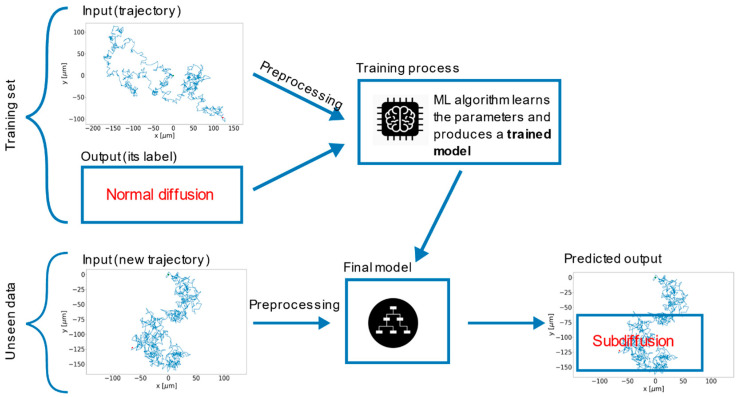
Workflow of trajectory classification by supervised machine learning. A training set of labelled trajectories is used to train the model. The trained model is then used to classify unlabeled (unseen) data. Preprocessing can consist of extracting relevant features. Reproduced with permission from [127]. Copyright (2020) by The American Physical Society.

Classification is one of the main tasks that machine learning (ML) can solve in a wide range of applications today, and the field of trajectory analysis in SPT also benefits from the recent explosion of this type of approaches. In the following, we give examples of the numerous methods based on this branch of artificial intelligence (AI) that have been introduced to overcome the limitations of previous strategies. Figure 6 shows a general example of trajectory classification by supervised ML, a type of algorithm particularly used for these purposes. It involves training a model on a labeled dataset, in which each training example is paired with an output label; the model learns to make decisions based on this input–output mapping.

Wagner et al. developed a random forest classifier (a supervised approach that exploits an ensemble of decision trees [128]) to segment and classify particle trajectories into four main motion types (confined, normal, directed, anomalous diffusion). They used the following nine features to characterize the trajectories: the anomalous exponent, the asymmetry, the efficiency (related to the ratio of the squared net displacement to the sum of the squared step displacements), the fractal dimension, a measure of gaussianity for the displacements, a measure of kurtosis (calculated using the positions projected on the dominant eigenvector of the radius of gyration tensor), a mean squared displacement ratio between two different time lags, the straightness (related to the ratio of the net displacement to the sum of step lengths), and the trappedness (probability of trapping) [89]. The method, after training on simulated tracks, was able to infer the motion type and its parameters for each segment under different experimental conditions. The developed software is freely available as an ImageJ plugin (TraJClassifier, available at [90], see also Table 1).

An ML random-forest-based method was also developed by Muñoz-Gil et al. to classify tracks showing normal or anomalous (sub- or super-diffusion) motion with the calculation of the anomalous exponent [129]. The approach also worked for short tracks and in the presence of noise, and the authors showed its ability to transfer learning to data generated with theoretical models not included in the training set.

**Figure 7:**
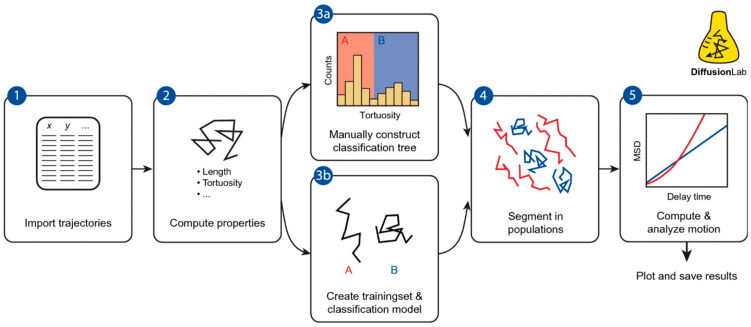
Workflow of DiffusionLab trajectory analysis. (**1**) Trajectories are imported; (**2**) a set of trajectory properties is calculated; (**3**) a classification model is constructed either manually (**3a**) or by machine learning (**3b**); (**4**) trajectories are classified with the constructed classification model and pooled into populations with similar behavior; (**5**) MSD analysis is performed. Reproduced with permission from [91] © The Author(s) 2022.

Another software package developed for the analysis of SPT data under difficult conditions, i.e., the presence of short trajectories and heterogeneities, is DiffusionLab [91] (Figure 7, Table 1). In this package, after importing the trajectories, a set of features is calculated for each of them. The software includes some predefined features, and user-defined ones can be added by creating new code files. The predefined features are the number of points (number of consecutive localizations), length (sum of all the individual displacements), radius of the minimum bounding circle (radius of the smallest enclosing circle that can be drawn around the localization coordinates, describing their spatial extension), distance between the center of the minimum bounding circle and the center of mass (describing how homogeneously points are spatially distributed), elongation (weight of the first principal component of localization coordinates), elongation angle (direction of the first principal component of localization coordinates), entropy (measurement of spatial randomness), and tortuosity (the ratio of the distance between the start and end points versus the length of the track, describing start-to-end directionality) [91,130]. A classification model is built either manually (by setting user-defined thresholds on the features) or by machine learning through a hierarchical classification tree (other supervised machine learning tools available in MATLAB can also be used). In the case of machine learning, the thresholds and trajectory features to be considered are selected automatically after training the model on a suitable dataset (up to five classes allowed); the classification model is used to group tracks into populations with similar behaviors. The analysis of the motion, useful to extract parameters characterizing each group, is then performed either on each track or on each group of tracks, considering the following: time-averaged MSD analysis can be performed on each track if they are sufficiently long; however, if the tracks are too short, it is averaged over multiple tracks within the same group to obtain more robust estimates of motion parameters. The software requires MATLAB and is freely available from [131]. DiffusionLab supports importing tracks from three SPT software—DoM (Image-J plug-in), Localizer (Igor Pro plug-in), and COMSOL Multiphysics (a commercial software for simulations) [130]. Although the developers state that support for importing other formats can be requested, a useful improvement might be the ability to automatically import from some of the most widely used SPT software for life sciences, such as u-track and TrackMate [10,132].

Pinholt introduced a general approach for SPT analysis (called “diffusional fingerprint”) that allows for the extraction of diffusional patterns of tracks, independently of the underlying diffusion type [133]. It is based on a set of 17 descriptive features, providing the advantage of not requiring a priori assumptions about the type of movement, unlike model-based analysis. Classification is achieved by a pattern recognition algorithm based on ML, which trains a logistic regression model. The authors demonstrated that training and prediction can be performed on experimental data, eliminating the need for pre-training on simulated data and the assumption of correspondence between simulations and experiments. To train the model to understand when two processes are dissimilar, different experimental conditions were used. The label in the training on experimental data corresponded to the different observed experimental conditions. The distributions of features and the ranking of the relevance of the features were used in the application of the trained model to decide whether two diffusion processes were different and to infer important diffusion differences in microscopic motion. The method has been tested on a variety of simulated and experimental scenarios, showing great flexibility and applicability in many cases. It accurately assigned diffusional characteristics regardless of the type of dynamics, using the same 17 characteristics in all cases, providing a general way of mapping different phenomena in a common space.

Dosset et al. developed an approach based on a back-propagation neural network (BPNN, a type of neural network that uses the back-propagation algorithm for training, where the term “back-propagation” refers to the way the model adjusts its weights based on the error rate obtained in the previous iteration [134]), which takes in input the MSDs calculated on a sliding window along the trajectory [135]. After training on simulated trajectories exhibiting Brownian, confined, and directed diffusion, the algorithm can distinguish the three types of motion even within a single trajectory.

**Figure 8:**
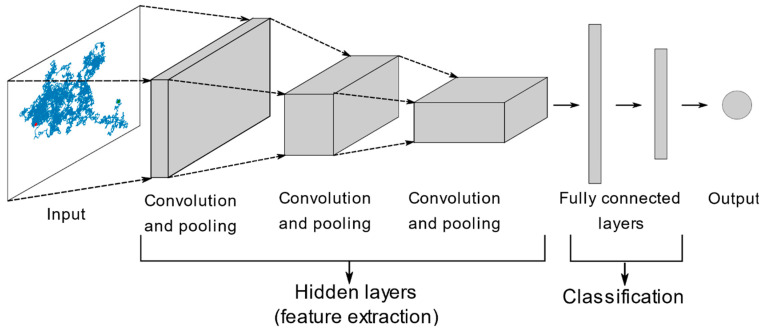
Sketch of the architecture of a convolutional neural network (CNN). The hidden layers automatically extract relevant features from the trajectories; they include convolutional layers (which apply convolution operations to the input data to extract local features and capture spatial hierarchies) and pooling layers (which reduce the spatial dimensions of the feature maps and thus help in reducing the number of parameters and the computational complexity and in controlling overfitting, retaining the most important information while discarding non-essential details). The extracted features are used by the successive classification part of the algorithm based on fully connected layers in which each neuron in a layer is connected to every neuron in the previous layer, allowing the network to combine the extracted features. Reproduced with permission from [136]. Copyright (2019) by The American Physical Society.

Kowalek et al. proposed a deep learning convolutional neural network (CNN, Figure 8) for track analysis in SPT [136]. Unlike some other ML methods, including the ones cited above, which require user-defined features, deep learning approaches require no data preprocessing and extract the relevant features automatically, without human intervention. CNN excels at tasks involving structured grid data, particularly images, by leveraging convolutional and pooling operations to extract and learn the spatial hierarchies of the features from the input data [137]. Their use of time series and trajectory analysis is an emerging field since trajectories can be encoded into a 2D grid (image-like structure) [138,139]. Kowalek et al. compared the deep learning CNN with classical ML methods (random forest and gradient boosting, both based on decision trees) for the identification of different motion modes in SPT. All models were trained with simulated data (which included normal, directed, confined, and anomalous diffusion). CNN showed a slightly higher accuracy than the other considered methods in most cases, but it required longer processing times. The authors found that, as expected, the ML methods generally failed to classify correctly motion models that were not included in the training data, even if they produced similar diffusion trends, such as different types of confined diffusion, with CNN being the worst in this respect. The study was carried out considering only homogeneous motion within tracks, i.e., a single mode of motion per track; the authors speculated that the CNN approach might show more advantages over the other ML models in the presence of heterogeneities within single tracks, since most of the human-defined features typically used are based on MSD estimates, which are the worst for short track segments.

Based on the observation made in the last discussed work, in particular that (i) all models failed to generalize the knowledge to the unseen (not present in the training data) types of motion and (ii) that traditional ML is cheaper in terms of time and computational resources and more interpretable than deep learning, Janczura et al. improved the random forest and gradient boosting methods, proposing a new set of features that characterize the tracks and can be used for their classification [127]. For the choice of the features, they considered the study by Weron et al. ([113], discussed above), in which the author compared classical hypothesis testing on statistics obtained by MSD, maximum excursion and p-variations, and found that none was the best in all cases thus suggesting a combined approach. The new set of features chosen by Janczura et al. thus included quantities based on the diffusion coefficient, the anomalous exponents, the maximum distance, and the p-variations. This set was used to train the ML models and to classify trajectories. They showed improved knowledge transferability and, very importantly, that choosing the right features is a crucial point, greatly affecting the accuracy of the results. Moreover, knowing the most and least important features helps to retain only those that actually drive the results and to omit the less informative ones, in order to reduce dimensionality and make the model faster. *p*-variation turned out to be the most informative feature, while the diffusion coefficient was the least, with the removal of the latter changing the relative importance of the remaining features.

Granik et al. were interested in resolving processes that produced different kinds of anomalous diffusion, even starting from short trajectories. They presented a deep learning approach based on a set of convolutional neural networks (trained on simulated data) to classify trajectories by three types of diffusion (Brownian and two kinds of anomalous diffusion, i.e., FBM and CTRW) and to infer motion parameters. Compared to traditional MSD-based analysis, they demonstrate greater accuracy, the need for much less data to achieve the same precision, a greater ability to extract information from even very short tracks, and an increased robustness to noise [140].

One of the main difficulties of supervised ML approaches is finding a good training set, i.e., sets of trajectories as similar as possible to the experimental ones to be analyzed; the population in this training set should be quite high for the classification to be correct. Indeed, as noticed above, often the algorithm is wrong on trajectories that are somehow different than the ones used in the training set, even if the underlying model should be the same. Often the training set is obtained by simulation, but it is not always easy to include all the elements defining the experimental situation, and often each experiment (e.g., biomolecules motion in different cell types or with different treatments) should be considered unique. Another approach is performing a careful traditional analysis on a subset of trajectories, checking the results one by one, and using the ML approach to make automatic classification and parameters extraction on the remaining trajectories, and/or to refine the parameters (like the thresholds) for the classifications based on features statistics. This drawback could cast doubts on the comparisons of power and accuracy between ML and more traditional approaches, since the comparisons are usually made on the sets used to train the ML algorithm, while the traditional methods could also be more easily adaptable to completely different datasets without the need for training (but often requiring careful supervision by the researcher, who could introduce some bias). A possible solution can arise from unsupervised ML approaches, where the objects to be classified are grouped only according to their similarities, without being given any labels. In this case, the characterization of the different classification groups has to be made a posteriori¸ introducing therefore difficulties in the interpretation of the results and again in their applicability to different datasets. To solve this last issue, the algorithm could be applied again on the whole dataset if some data are added for making a comparison, but this could change the established classes.

An example of an unsupervised machine learning-based classification method for single-particle trajectories is pEM (perturbation expectation-maximization) [88] (Table 1). It uses a system-level likelihood function (a Gaussian mixture model of multivariate Gaussians) to collectively describe a population of trajectories with different diffusion coefficients and localization noise. Convergence to the maximum of the likelihood function is performed by a novel algorithm (perturbation expectation-maximization) that uses iterative perturbations on the likelihood function. pEM extracts the number of diffusive states with their properties from a population of tracks and statistically classifies each track into a diffusive state. The method accounts for the experimental correlation between particle displacements due to nearest-neighbor displacements and to localization noise. pEM was tested on simulated data and experimental ones, the last ones obtained by tracking proteins of the Rho GTPases families, finding six diffusive states, with the number of states conserved across different proteins with variability in the population fractions. The performance depended on the length of the trajectories, where shorter trajectories led to a broadening of the diffusivity distribution of each state and made it difficult to distinguish between the states; and longer trajectories increased the probability of transition between different diffusive states. The first version of pEM had the following two main limitations: it assumed normal motion and no transition between diffusive states. A successive version of the algorithm (pEMv2) was developed to overcome them [87]. pEMv2 also allowed for the analysis of non-normal diffusion modes (it becomes a model-free approach with no prior assumption about the nature of the motion) and could deal with motion transitions by splitting trajectories into shorter segments of constant length, each segment being associated with a diffusive state. The optimal segment length can be chosen by trying various values and selecting the condition yielding the highest likelihood. Indeed, too short a length may lead to misclassification, but too long a length may cause the inclusion of an increasing number of transitions between different types of movement. For example, pEMv2 has been applied to SPT of histone H2B and transcription factors, revealing two states with dynamic switching between them and populations influenced by activation conditions [75]; and to B-cell receptors as well, revealing eight distinct states, again correlating with activation states [141].

In 2021, a competition was held to evaluate the methods for detecting and characterizing anomalous diffusion—the Anomalous Diffusion (AnDi) Challenge [142]. The participating teams used their approaches to solve the following three tasks: determination of the anomalous diffusion exponent, which entailed the clasnosification of the diffusion mode; track segmentation with the identification of change points within tracks, where the anomalous exponent and the diffusion mode changed; and the determination of the motion model and the anomalous exponent for each segment. The third task turned out to be the most difficult, even in the relatively simple condition of a single change-point per track, and, in fact, far fewer teams participated in this task than for the first two. The author observed that the traditional MSD approach had several limitations, especially in the case of short and noisy trajectories, heterogeneous motion, non-stationarity, non-ergodicity; furthermore, distinct kinds of physical processes can produce the same scaling exponent, making it impossible to distinguish the underlying model. Most of the models used in the competition outperformed the performance of the traditional MSD analysis. The best models were based on machine learning (ML) approaches, showing that ML can extract more information than classical statistics. However, the authors noted that classical statistics can still be helpful, especially because of the black-box nature of ML.

Starting from the results of the AnDi competition, Seckler et al. published a recent perspective focusing on the improvement in the explainability and interpretability of ML approaches that were successful in the challenge. In particular, they investigated the possibility to extract a set of statistical features to be used in the ML algorithms instead of the raw position data, and then to determine uncertainty estimates [143]. Notably, the authors observed that the analysis with classical statistical methods was still necessary to assess the validity of ML results and to prepare training data as possible models, and that not including the noise in the training data can lead to inaccurate predictions.

The results of the AnDi challenge were also exploited as a starting point by Manzo to develop a method based on Extreme Learning Machine (ELM) applied to a set of engineered features (AnDi-ELM [144]). ELM is an algorithm for single hidden layer feedforward neural networks with a much faster learning speed than other ML methods [145,146]. The features used in AnDi-ELM are based on estimators from classical statistics, i.e., the scaling exponent of the moments of the displacement distribution, the time-correlation of displacements, and the cumulative sum of squared displacements. With minor modifications to the entry features, the method was able to cope with the three tasks of the challenge, although it performed better on the classification task than on the regression with anomalous exponent estimation. The overall performance was considered satisfactory, especially as the method allowed for reduced training time, low computational cost, and an undemanding implementation compared to other ML approaches. Thus, the author suggested that it may be particularly useful for preliminary screening prior to further and more time-consuming evaluations.

The AnDi challenge stimulated the development of another ML-based method for characterizing anomalous diffusion called WADNet, published by Li et al. [147]. This is a deep neural network based on a modified version of WaveNet (the latter being a deep neural network developed for the generation of raw audio) combined with Long Short-Term Memory (LSTM) networks, a particular type of recurrent neural network used specifically for the classification of sequential data thanks to its ability to capture historical information. The method was applied to the AnDi challenge dataset and proved to outperform the best methods found in the original AnDi challenge for the first and the second tasks (inference of the anomalous exponent and classification of the diffusion model). The main architectures of WADNet are almost the same for the different tasks, with modifications concerning the input and output dimensions of the network.

Verdier et al. proposed the use of graph neural networks, a class of deep learning methods that use descriptions of data by graphs [148]. In their approach, a vector of features was associated with each position in the trajectory and a graph was associated with each trajectory. Training was performed on simulated data. The strategy was tested on the anomalous motion modes proposed in the AnDi challenge, and it showed the ability to infer the motion model and the anomalous exponent.

A new competition has just been launched from the same group at the beginning of 2024, The 2nd Anomalous Diffusion Challenge, which aims to compare methods for detecting motion changes in single-particle motion [149]. It proposes an analysis of both raw videos and trajectories; the aim is to identify transient changes of the generalized diffusion coefficient and/or anomalous exponent, transient interactions, transient confinement, transient immobilization. The challenge is organized into the following two tasks: the first one is to infer the simulated model, the number of states, the time spent in each state and then, for each state, the mean and standard deviation of the distribution of the generalized diffusion coefficients and of the anomalous diffusion exponent. The second task is to find the change points for each trajectory and then, for each segment, to find the type of motion, the generalized diffusion coefficient and the anomalous diffusion exponent. The competition is still open [150].

## 6. Discussion and Conclusions

We reviewed various analysis methods available today for studying trajectories obtained by SPT, along with the different advantages, potential, and open challenges for each of them.

Traditional MSD-based analysis remains a well-established and valid tool, important for historical reasons and easy to apply and interpret. Its use in SPT has allowed us to obtain important insights into molecular dynamics in different types of applications. However, as we have discussed, its application requires careful consideration of some aspects that have emerged over time and that have not always been taken into account, particularly in relation to the impact of experimental uncertainties. Several studies highlighted the limitations of analyses based on MSD, related to the presence of heterogeneities within tracks and to data consisting of a few noisy and short trajectories. Furthermore, conventional MSD analysis often fails to characterize anomalous motion types because different physical mechanisms can give rise to the same scaling behavior, making MSD incapable of distinguishing the different underlying physical diffusion processes.

Many alternative approaches have been developed for the analysis of SPT trajectories. Classical statistics have been based on parameters other than displacements, such as angular or velocities distributions, mean–maximum travelled distances, passage times, and p-variations.

A class of methods has been introduced for the analysis of transient behaviors that are masked in MSD analysis, e.g., approaches based on the detection of transient confinement or analysis within sliding windows. Indeed, amongst the important objectives in SPT trajectory analysis, there are segmentation and classification, as trajectories can show heterogeneous motions, reflecting the heterogeneity of the environment, the presence of interactions or other processes. Hidden Markov models have been exploited quite widely for this task; the state is usually described by a diffusion coefficient and transitions between states with different diffusion coefficients are investigated. A challenge in this area concerns the transitions between states with motions other than pure Brownian, for which a description of states based simply on a diffusion coefficient is not sufficient. The a priori selection of a fixed number of states has also been a limitation of HMM models in some cases, and this has been addressed and overcome in some of the studies we have discussed above.

Finally, we described methods based on machine learning. Classification is one of the main tasks approached with these kinds of methods nowadays. Therefore, in recent years, many algorithms based on machine learning have been applied to trajectory classification. They use different strategies, from more traditional ML, such as decision trees, to deep learning. Most of these methods are based on a set of track features to be used as input of the ML algorithm that classify the tracks. Different features have been exploited by the different methods as follows: they can be defined in the development of the model, can be expanded by the user in some cases, or can be automatically extracted in the case of deep learning. Classification is performed amongst different motion types or in a feature space. Identifying the best set of features is a critical issue and can greatly influence the results. Typically, features consisting of variables calculated starting from the MSD and other classical statistics have been used. Often ML approaches have been described to be more powerful (e.g., applicable to noisier or shorter trajectories) and accurate than the ones based on more classical statistics, but there could be doubts driven by the fact that usually these comparisons are made on data of the same type used to train the ML algorithms; indeed, as discussed above, ML-based algorithms often fail when applied to different datasets, or when different sources of noise are included. Aside from these difficulties in transfer learning, also due to the needed or possible training on simulated data, several other challenges have been reported in the field of machine learning, such as lack of interpretability, and high time and computational costs for training and analysis.

We also reported some freely available software for analyses based on machine learning, exploitable even by users without programming experience.

Despite the numerous available analysis methods, the characterization of anomalous diffusion and track segmentation are active fields, as we have highlighted in the discussion of recently launched competitions. Challenges such as AnDi are useful to compare available methods, to stimulate the development of new ones, and to establish common datasets.

In conclusion, the analysis of SPT trajectories encompasses a wide range of methods that are constantly evolving. The traditional MSD is still used and useful, and no other one is as widespread and established; however, alternative methods can now help to overcome its limitations. A successful strategy may use a combined approach that takes advantage of more methods, such as a combination of classical statistics and machine learning. This review can be a useful guide to identify the methods suitable for the situation of interest, as well as an inspiration for new ideas and improvements.

## Figures and Tables

**Table 1:** Freely available software for SPT analysis. The columns report the name of the software, the type of analysis implemented, the types of motion considered, the programming language in which it is implemented, whether localization error is considered or not, the number of mobility states that can be considered, the experimental (if available) application reported in the software publication, and the reference for the software or where it is described.

Software	Method	Motion Models	Implementation	Localization Error	Number of States	Studied Proteins	Ref.
Spot-On	fitting of the empirical displacement distribution	Brownian	web interface, Matlab, Python	yes	2 or 3	H2B, CTCF, NLS, Sox2	[76,77,78,79,80]
Saspt	Bayesian analysis	model free	Python	yes	found by the analysis	RARA, H2B	[82,83,84]
vbSPT	HMM	model free	Matlab	no	found by the analysis	RNA-binding protein Hfq	[85,86]
pEMv2 ^1^ (https://github.com/MochrieLab/pEMv2 (accessed 6 August 2024))	machine learning (perturbation expectation-maximization)	model free	Matlab	yes	found by the analysis	Rho GTPases (version 1), simulations (version 2)	[87,88]
TraJClassifier	random forest	normal diffusion, subdiffusion, confined diffusion, directed motion	imageJ plugin	yes	4	fluorescent nanoparticles	[89,90]
DiffusionLab	manual or machine learning classification	model free	Matlab	yes	up to 5	simulations	[91]
iHMMSPT	infinite HMM	Brownian	Matlab	no	found by the analysis	B-cell receptor	[92,93]

^1^ See the main text for the explanation about the version 1 of the software.
